# Dysregulated Cholesterol Metabolism and Its Impact on the Tumour Immune Response in Colorectal Cancer

**DOI:** 10.1111/jcmm.70789

**Published:** 2025-08-10

**Authors:** Chunyu Zhang, Zhiwei Miao, Yan Xu, Xingchao Zhu, Tongguo Shi

**Affiliations:** ^1^ Department of Gastroenterology Zhangjiagang TCM Hospital Affiliated to Nanjing University of Chinese Medicine Zhangjiagang China; ^2^ Jiangsu Institute of Clinical Immunology The First Affiliated Hospital of Soochow University Suzhou China; ^3^ Department of Gastroenterology The Yancheng School of Clinical Medicine of Nanjing Medical University (Yancheng Third People's Hospital) Yancheng China

**Keywords:** cholesterol metabolism, colorectal cancer, immune responses, therapy

## Abstract

Colorectal cancer (CRC) ranks among the primary causes of cancer deaths worldwide. Cholesterol metabolism, tightly regulated through biosynthesis, uptake, efflux and conversion, plays a crucial role in CRC development and progression. Dysregulated cholesterol metabolism in cancer cells alters the tumour microenvironment (TME), promoting tumour progression and immune escape. Recent studies highlight the relationship between cholesterol metabolism and the body's immune reactions, showing that cholesterol and its metabolites can modulate immune cell function, driving pro‐tumorigenic or anti‐tumour effects. Targeting cholesterol metabolism has shown promise in enhancing immunotherapy outcomes, such as inhibiting specific enzymes or transporters to improve T cell infiltration and activation. In this review, we provide a comprehensive overview of the functions of cholesterol and its metabolites in the immune response and the latest advances in CRC therapy targeting cholesterol metabolism.

## 1 Introduction

Colorectal cancer (CRC) is one of the most prevalent and deadly malignancies worldwide [1, 2]. According to the 2025 cancer statistics, it is projected that there will be approximately 154,270 new cases of CRC and around 52,900 CRC‐related deaths in the United States [3]. A combination of genetic, environmental and lifestyle factors, including diet, influences the development of CRC [4, 5, 6]. High dietary cholesterol intake, in particular, has been connected to a greater likelihood of developing cancer and might impact the survival of those with cancer [7, 8]. Notably, studies suggest that there is a correlation between increased serum cholesterol levels and a greater risk of CRC [9]. However, the precise relationship between cholesterol and CRC, as well as the underlying mechanisms, remains incompletely understood.

Cholesterol, a vital component of mammalian cell membranes, plays a crucial role in maintaining membrane integrity and fluidity [10, 11]. Its metabolism is tightly regulated through procedures such as biosynthesis, assimilation, release and conversion [12, 13]. Preclinical and clinical studies have shown that high‐fat diets and hypercholesterolaemia contribute to tumorigenesis and cancer progression by activating metabolic pathways like the Hedgehog pathway and mTORC1 [14, 15, 16]. Cholesterol and its byproducts function as signalling agents that drive tumour progression, including in CRC [17, 18, 19].

Aberrant cholesterol metabolism in cancer cells alters the tumour microenvironment (TME), promoting tumour progression and impairing treatment efficacy [20, 21, 22]. Cholesterol metabolism also influences tumour‐infiltrating immune cells, modulating their anti‐tumour or pro‐tumour effects [23]. Recent progress in comprehending the relationship between cancer metabolism and the immune system has led to promising combination strategies targeting cholesterol metabolism to enhance immunotherapy. For example, inhibiting the cholesterol metabolism enzyme cholesterol‐25‐hydroxylase (CH25H) can reverse the immunosuppressive function of tumour‐associated macrophages (TAMs) and enhance T cell infiltration and activation, improving the efficacy of immune checkpoint inhibitors like anti‐PD‐1 antibodies [24]. Similarly, targeting Proprotein Convertase Subtilisin/Kexin Type 9 (PCSK9), a cholesterol metabolism regulator, through gene editing or neutralising antibodies, can increase MHC I expression on tumour cells, enhancing T cell‐mediated tumour killing [25].

Despite these advances, current immunotherapy for CRC remains limited due to the complexity of the TME, tumour heterogeneity and immune escape mechanisms [26, 27, 28]. This review highlights the role of cholesterol metabolism in cancer immunity and explores the potential of targeting cholesterol metabolism to improve immunotherapy outcomes in CRC.

## 2 The Equilibrium of Cholesterol Metabolism

**FIGURE 1:**
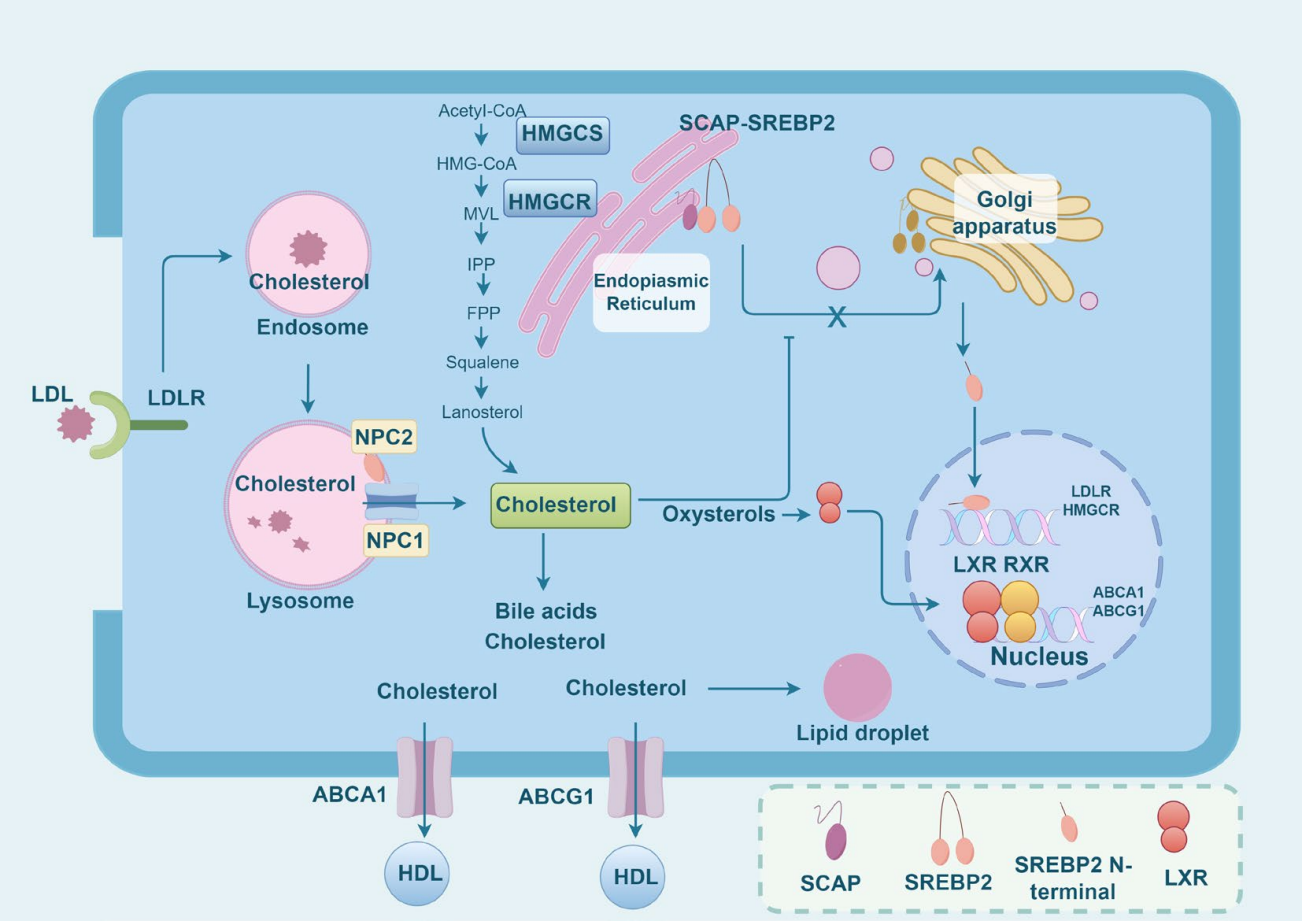
The key biochemical process of cholesterol homeostasis in cells. This process encompasses the intracellular synthesis of cholesterol, the exogenous uptake of cholesterol, and the efflux of intracellular cholesterol. The graphic was created by Figdraw (www.figdraw.com).

Intracellular cholesterol homeostasis refers to the dynamic balance maintained by cells through finely tuned regulatory mechanisms, involving the synthesis, uptake, storage, utilisation and efflux of cholesterol [12]. This process is crucial for the integrity of cell membranes, signal transduction, hormone synthesis (such as steroid hormones) and lipid metabolism [29]. To better understand the mechanisms underlying cholesterol homeostasis, it is essential to introduce both the intracellular synthesis and the exogenous uptake of cholesterol (Figure 1) [17].

Cholesterol synthesis begins with acetyl‐CoA and proceeds through a series of enzymatic reactions that ultimately produce cholesterol. To initiate this process, the enzyme acetoacetyl‐CoA thiolase facilitates the condensation of two acetyl‐CoA molecules to create acetoacetyl‐CoA, which then interacts with an additional acetyl‐CoA molecule in the presence of HMG‐CoA synthase (HMGCS) to form 3‐hydroxy‐3‐methylglutaryl‐CoA (HMG‐CoA) [12]. Then, under the influence of HMG‐CoA reductase (HMGCR), HMG‐CoA is converted to mevalonate (MVL) through reduction by NADPH [30]. Subsequently, mevalonate undergoes a cascade of enzymatic transformations that sequentially yield 3‐isopentenyl pyrophosphate, farnesyl pyrophosphate, squalene and lanosterol, culminating in the biosynthesis of cholesterol [31]. Furthermore, through a series of reactions such as oxidation, demethylation and reduction, lanosterol is converted into cholesterol. These reactions involve various enzymes, including cytochrome P450 family enzymes and reductases [32].

Additionally, exogenous cholesterol uptake refers to the process by which cells acquire cholesterol from the external environment, primarily through the uptake of low‐density lipoprotein (LDL) particles mediated by the LDL receptor (LDLR) [33]. Meanwhile, cholesterol efflux is mainly mediated through two pathways: ATP‐binding cassette transporter A1 (ABCA1)‐mediated cholesterol efflux and ATP‐binding cassette transporter G1 (ABCG1)‐mediated cholesterol efflux [34, 35]. Moreover, the intracellular synthesis of cholesterol is tightly regulated by feedback mechanisms to ensure dynamic balance in cellular cholesterol levels. The regulation of HMGCR gene expression is mediated by Sterol Regulatory Element‐Binding Protein 2 (SREBP‐2) [36]. When cholesterol within the cell is scarce, SREBP‐2 is activated and migrates to the nucleus, enhancing the expression of HMGCR. In contrast, elevated cholesterol levels lead to the inhibition of SREBP‐2 activity. Finally, the activity of HMGCR is also regulated by phosphorylation and dephosphorylation [12].

In summary, cholesterol metabolism homeostasis is a highly regulated process involving multiple organelles and signalling pathways. Its dysregulation is linked to various diseases, positioning it as a crucial target for therapeutic and drug development efforts. By regulating cholesterol synthesis, uptake and efflux, intracellular cholesterol levels can be balanced, promoting overall metabolic health. Understanding these mechanisms offers valuable insights for developing therapeutic strategies for cholesterol‐related diseases. Additionally, this balance extends its influence to critical biological processes like tumour–immune interactions in cancer.

## 3 Cholesterol Metabolism Modulates Tumour–Immune System Interactions in CRC

**FIGURE 2:**
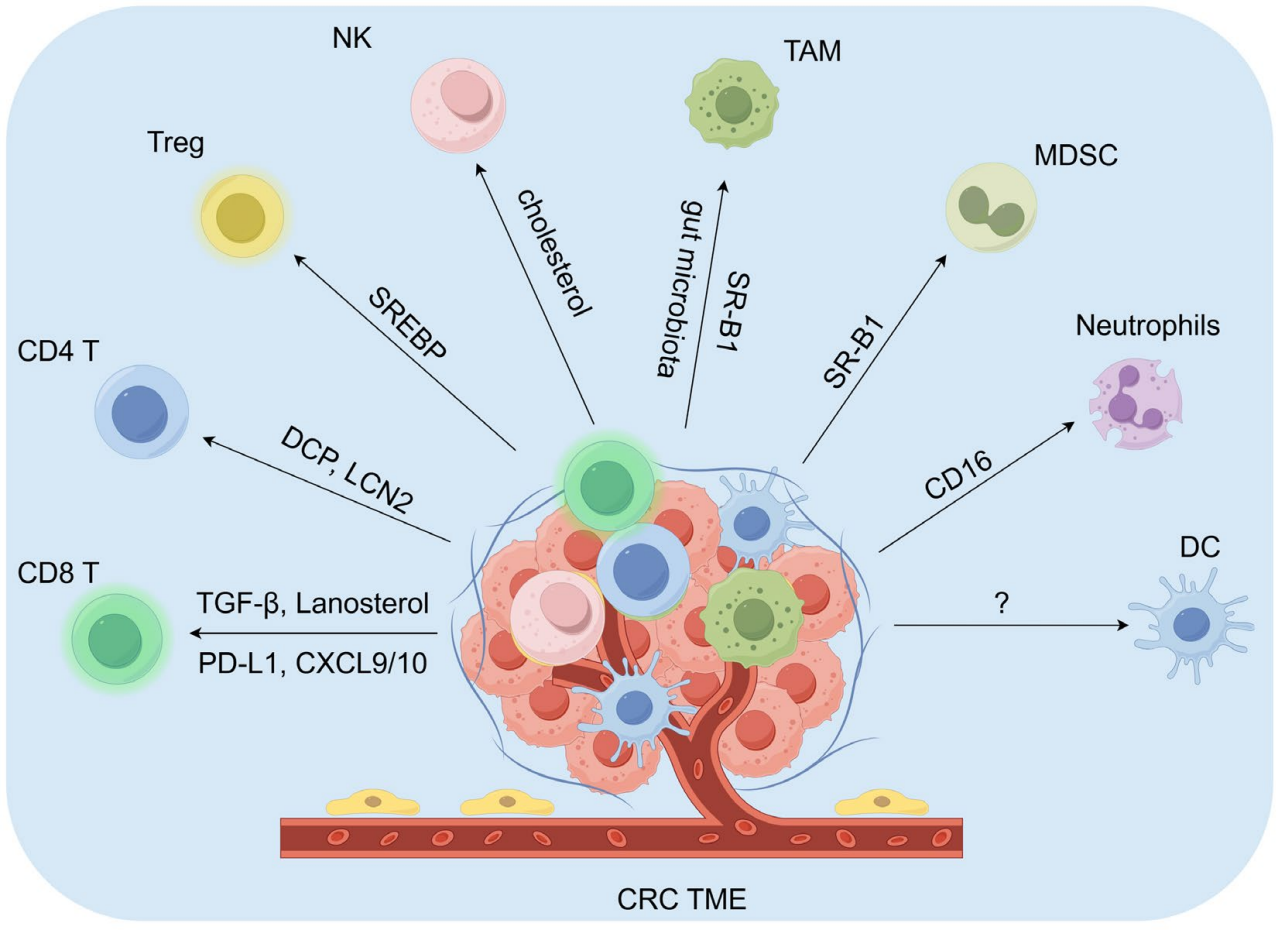
The effects of cholesterol metabolism on immune cells in CRC TME. The graphic was created by Figdraw (www.figdraw.com).

Within the TME, cholesterol and its derivatives are important in determining whether immune cells exhibit anti‐tumour or tumour‐tolerant behaviours [17]. Thus, gaining a more profound insight into cholesterol metabolism in tumours could unveil new opportunities for immune sensitisation and therapeutic intervention. Now, we will touch on the roles of cholesterol metabolism in CRC via the regulation of immune cells, such as CD8^+^ T cells, CD4^+^ T cells, regulatory T (Treg) cells, natural killer (NK) cells, TAMs and MDSCs (Figure 2).

### 3.1 CD8 ^+^ T Cells

**FIGURE 3:**
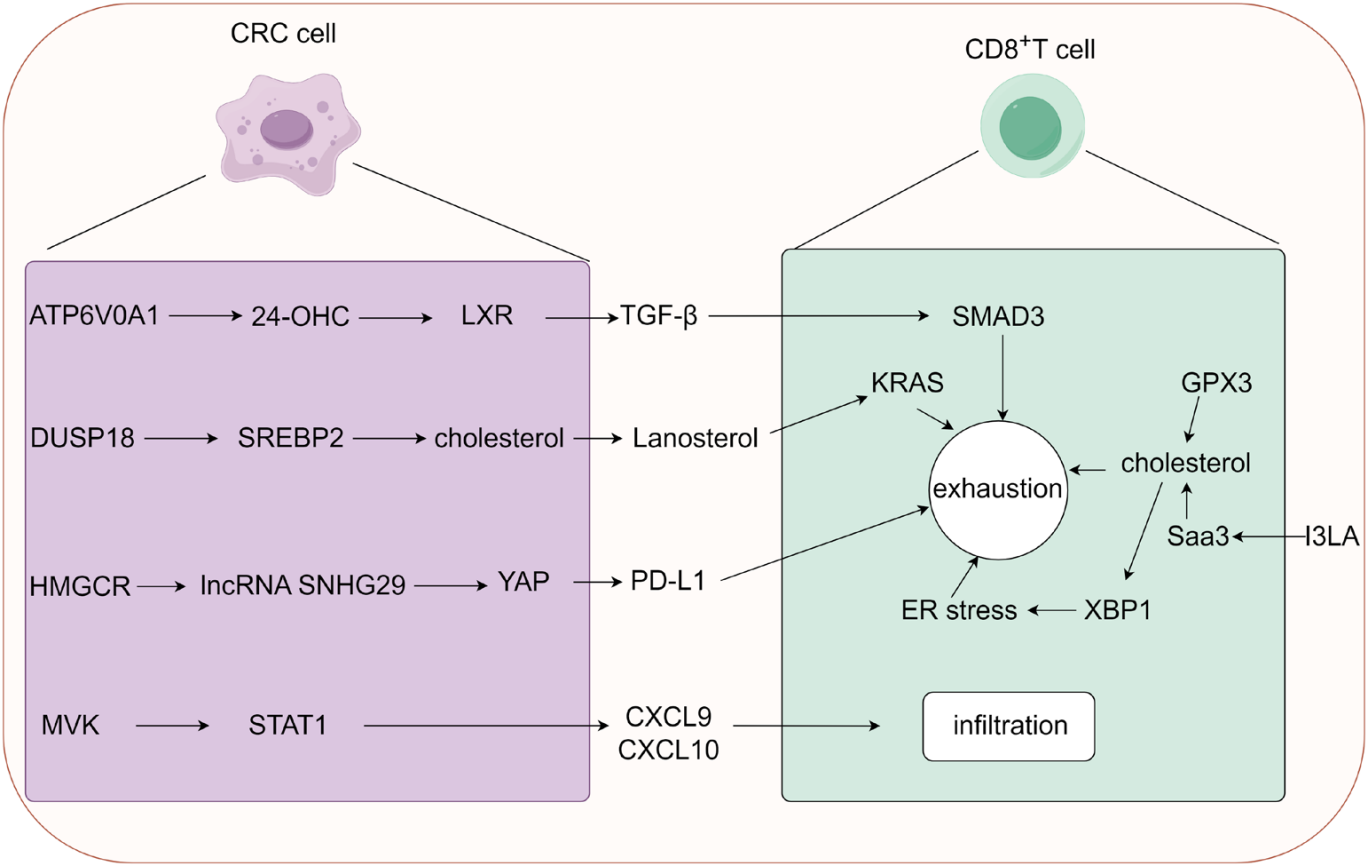
Roles of cholesterol homeostasis in modulating CD8^+^ T cell function in CRC. The graphic was created by Figdraw (www.figdraw.com).

CD8^+^ T cells, known as cytotoxic T cells, have the ability to directly kill tumour cells by discharging perforin and granzymes [37]. Clinical studies have indicated that CD8^+^ T cells infiltrating at high levels within tumours are positively correlated with patient survival rates [38]. Importantly, the functional status of CD8^+^ T cells in CRC is controlled by numerous intricate factors, such as cholesterol metabolism (Figure 3).

For patients with stage II/III CRC, there was a positive correlation between HDL cholesterol (HDL‐C) levels and the presence of CD3^+^ T cells, CD8^+^ T cells and iNOS^+^ cells, which were associated with a favourable prognosis [39]. Additionally, cholesterol's immunosuppressive arsenal extends to ER stress—overload in CD8^+^ T cells activates X‐box binding protein 1 (XBP1), upregulating exhaustion markers (PD‐1, TIM‐3). Depleting cholesterol or alleviating ER stress restores anti‐tumour vigour [40, 41]. The research by Zhang et al. further indicated that administering *Lactobacillus plantarum* L168 and its metabolite, indole‐3‐lactic acid (I3LA), mitigated intestinal inflammation, decreased colorectal tumour growth, and resolved gut dysbiosis. I3LA reduces the expression of Saa3, related to cholesterol metabolism in CD8^+^ T cells, through transcriptional inhibition and chromatin accessibility alteration, enhancing the performance of tumour‐infiltrating CD8^+^ T cells [42]. Additionally, glutathione peroxidase 3 (GPX3), a prognostic marker associated with cholesterol metabolism, correlates with CD8^+^ T‐cell exhaustion in CRC by modulating cholesterol levels within CD8^+^ T cells [43]. Collectively, these studies suggest that cholesterol metabolism in CD8^+^ T cells significantly influences their function in CRC.

Aberrant cholesterol metabolism in CRC cells exerts a significant influence on CD8^+^ T‐cell exhaustion and infiltration. ATPase H+ transporting V0 subunit a1 (ATP6V0A1) drives cholesterol uptake through RABGEF1‐dependent endosome maturation, generating 24‐hydroxycholesterol (24‐OHC) to activate LXR‐TGF‐β1 signalling. This cascade paralyses memory CD8^+^ T cells via SMAD3, a vulnerability that can be reversed by the repurposed antiviral drug daclatasvir [44]. Similarly, deletion of dual specificity phosphatase 18 (DUSP18) unleashes CD8^+^ T‐cell activity by blocking lanosterol‐mediated KRAS suppression, revealing a metabolic checkpoint in immune evasion [45]. These findings highlight how cholesterol intermediates act as molecular puppeteers, influencing both tumour and immune cells.

Furthermore, mevalonate kinase (MVK), a key enzyme in cholesterol biosynthesis, dampens interferon responses in microsatellite instability (MSI) CRC by silencing Th1 chemokines such as CXCL9/10. Knockout of MVK reinvigorates CD8^+^ T‐cell infiltration [46], while the HMGCR inhibitor simvastatin disrupts PD‐L1 expression via inhibiting lncRNASNHG29/YAP axis, revitalising cytotoxic T lymphocytes [47].

In summary, the regulation of CD8^+^ T cell activity in CRC is linked to cholesterol metabolism, with key genes such as ATP6V0A1 and DUSP18 modulating their function through cholesterol trafficking and signalling pathways. Therapeutic strategies targeting cholesterol metabolism, such as MVK gene knockout and HMGCR inhibitors like simvastatin, have demonstrated potential in reactivating CD8^+^ T cells and boosting anti‐tumour immune responses in CRC. However, despite these findings, the cholesterol metabolic network is highly complex, and there may be many unknown regulatory mechanisms at play. Current research may only be scratching the surface, revealing just the tip of the iceberg. To enhance our understanding of cholesterol metabolism's role in CD8^+^ T cell function, further investigation is necessary, along with the development of improved therapeutic strategies.

### 3.2 CD4 ^+^ T Cells

In TME, CD4^+^ T cells exhibit a dual role in immune responses. On one hand, anti‐tumour responses can be enhanced by CD4^+^ T cells, including Th1 and Th17 cells, through the secretion of cytokines such as IFN‐γ and IL‐17, which activate the immune system. On the other hand, Tregs, a special subset of CD4^+^ T cells, may suppress anti‐tumour immune responses by secreting immunosuppressive cytokines such as IL‐10 and TGF‐β [48, 49]. Interestingly, recent research has underscored the crucial roles of cholesterol metabolism in controlling CD4^+^ T cell‐mediated immune responses in CRC, emphasising their potential use as targets in treatment.

At the heart of this interplay lies the role of distal cholesterol precursors (DCPs), intermediates lingering between lanosterol and cholesterol. These metabolites bind RORγt, a nuclear receptor pivotal for Th17 cell differentiation, driving pro‐tumorigenic immune polarisation. In microsatellite‐stable (MSS) CRC tumours, dysregulated cholesterol biosynthesis fosters DCP accumulation, crafting a Th17‐skewed TME that fuels malignancy [50]. This metabolic imbalance transforms CRC cells into covert manipulators, secreting DCPs to co‐opt immune defences into allies of tumour progression. Beyond direct metabolic reprogramming, cholesterol exerts its treachery through iron hijacking. Lipocalin‐2 (LCN2), overexpressed in CRC, disrupts CD4^+^ T‐cell iron homeostasis by accelerating iron efflux, triggering apoptosis and fostering a tumour‐permissive niche. Paradoxically, LCN2 simultaneously amplifies cholesterol metabolism in infiltrating T cells, further empowering CRC invasion [51], illustrating how lipid networks orchestrate both metabolic and immune dysfunction.

In sum, in the TME of CRC, the function of CD4^+^ T cells and the progression of CRC are affected by cholesterol metabolism, which regulates Th17 cell differentiation and iron metabolism, suggesting CD4^+^ T cells as possible therapeutic targets. However, many factors such as the complexity of the tumour microenvironment, unknown mechanisms of the cholesterol metabolic network, individual differences and tumour heterogeneity, challenges in clinical translation, and the complexity of multifactorial interactions may all limit a comprehensive understanding and effective utilisation of CD4^+^ T cell functions in CRC.

### 3.3 Treg Cells

Treg cells are critical for immune tolerance and exert immunosuppressive effects in the tumour microenvironment (TME), especially in colorectal cancer (CRC) [52, 53]. Targeting Treg cells in cancer therapy requires understanding their context‐dependent mechanisms. Metabolic reprogramming, including cholesterol metabolism, plays a key role in influencing Treg infiltration and function within the tumour microenvironment [54, 55]. In MC38 tumours, Treg cells show increased Pdcd1 expression, depending on SREBP activity linked to mevalonate metabolism‐driven protein geranylgeranylation. Blocking PD‐1 or SREBP signalling disrupts PI3K activation in intratumoural Treg cells [56]. Al‐Husein et al. found that statin treatment (HMG‐CoA reductase inhibitors) increases Treg infiltration in CRC tumours [57].

### 3.4 NK Cells

NK cells, key innate immune cells, are vital in the immune response against CRC [58, 59]. They directly lyse tumour cells by releasing cytotoxic granules, including perforin and granzymes, which induce apoptosis. Moreover, NK cells secrete cytokines like IFN‐γ to modulate immune responses and inhibit tumour progression [58, 59].

Liu et al. developed a cholesterol (C)‐to‐NK cell ratio (CNR) biomarker, highlighting the combined impact of cholesterol metabolism and inflammation on CRC outcomes. Elevated tissue CNR levels indicate a higher CRC risk, while lower levels are linked to better survival rates [60]. Moreover, CRC‐infiltrating NK cells often show lower intracellular cholesterol than normal CD56^+^ NK cells. Cholesterol reduction in NK cells by CD16^+^ neutrophils may decrease their cytotoxicity [61]. These findings suggest cholesterol metabolism's importance in NK cell function and CRC progression.

### 3.5 TAMs and MDSCs

In the TME, immunosuppressive cells, such as TAMs and myeloid‐derived suppressor cells (MDSCs), collaborate to inhibit antitumour immune responses and promote tumour progression and metastasis through the secretion of immunosuppressive cytokines, metabolites and chemokines [62, 63]. Consequently, strategies targeting these immunosuppressive cells have emerged as significant avenues for tumour immunotherapy research. For instance, inhibiting the immunosuppressive functions of TAMs or depleting MDSCs has shown promise in enhancing antitumour immunity [62, 63].

In CRC, a key factor in the modulation of TAMs and MDSCs is cholesterol metabolism. The cholesterol transporter SR‐B1 emerges as a dual‐faced architect of immune evasion. Knockdown of SR‐B1 in CRC models has been shown to reduce tumour burden by remodelling the TME. This involves reprogramming immunosuppressive cells (TAMs and MDSCs), modulating the expression of PD‐L1 and HLA‐B, and resetting cholesterol flux via LDLR and ABCA1 [64]. Strikingly, SR‐B1 silencing synergises with anti‐PD‐1 therapy, highlighting its combinatorial potential. 5‐Aza‐2′‐deoxycytidine (5Aza) functions as an inhibitor of DNA methyltransferase, regulating cholesterol buildup, p65 activation, and IL‐6 production by targeting ATP‐binding cassette transporter A9 (ABCA9). 5‐Aza causes primary mouse macrophages to polarise into an M1 phenotype, characterised by increased phosphorylated p65 and IL‐6 level, and suppresses CRC peritoneal carcinomatosis by modulating macrophage‐dependent T cell activation [65]. Meanwhile, research has demonstrated that the gut microbiota can utilise LDL‐C to rewire immune gene networks, bridging lipid metabolism and tumour progression [66]. This microbial‐metabolic axis underscores the complexity of CRC pathogenesis and suggests that targeting cholesterol metabolism and the gut microbiota may offer novel therapeutic strategies for CRC.

### 3.6 Neutrophils

Neutrophils, the most abundant type of white blood cells in the bloodstream, play a crucial role in the TME and exhibit a dual role in cancer dynamics, oscillating between pro‐tumour and anti‐tumour activities that significantly influence cancer progression [67]. Elevated neutrophil levels in CRC patients are associated with poor prognosis, and the neutrophil‐to‐lymphocyte ratio serves as an independent prognostic indicator [68]. Zhang et al. recently revealed that CRC tumour tissues specifically accumulate CD16^+^ neutrophils. These cells experience abnormal cholesterol accumulation due to CD16/TAK1/NF‐κB axis activation, which boosts scavenger receptors like CD36 and LRP1 for cholesterol intake. The region‐specific CD16^+^ neutrophils not only competitively inhibit NK cell cholesterol intake, disrupting lipid raft formation and antitumour signalling, but also release NETs to induce NK cell death [61].

In conclusion, cholesterol metabolism actively influences immune cell interactions rather than being a passive participant in CRC. Notably, dendritic cells (DCs), heterogeneous antigen‐presenting cells with dendrite‐like projections, are vital for capturing, processing antigens, and presenting them to T cells to initiate specific immune responses [69, 70]. In CRC, DCs have a dual role: they can activate T cells and induce immunological memory to fight tumours, but also secrete cytokines affecting tumour cell growth, proliferation and angiogenesis [71]. Cholesterol mobilisation is essential for DC maturation and the cancer immunogenic response [72, 73]. However, the regulatory effects of cholesterol metabolism on DC functions in CRC are still unclear and require further investigation. As research continues to explore the complex relationship between lipid metabolism and immune function, CRC treatment may evolve into a more precise and powerful approach by combining metabolic targeting with immune enhancement. Understanding these mechanisms is crucial for developing targeted therapies and improving patient outcomes.

## 4 Clinical Significance of Cholesterol Metabolism in CRC

The role of cholesterol metabolism has become crucial in the immune response and the progression of tumours in CRC [74]. This metabolic pathway is intricately linked to the pathogenesis of CRC through various mechanisms, including the regulation of cholesterol levels and the influence of lipid‐modulating agents [75]. Here, we reviewed the clinical significance of cholesterol metabolism in CRC (Table 1), underscoring its significance in cancer susceptibility, therapeutic outcomes, and as a possible therapeutic target.

**TABLE 1:** The clinical significance of cholesterol metabolism in CRC.

Study types	Cholesterol metabolites	Protein/gene	No. of patients	Clinical relevance	References
A prospective study	HDL‐C	/	593	Low HDL‐C level had a significantly higher risk of CRC	[76]
Clinical trial	/	CETP	99	CETP activity and mass were markers of elevated CRC risk	[77]
Randomised controlled trial	7‐OHC	/	1246	7‐OHC was associated with higher colorectal adenoma risk	[78]
Randomised controlled trial	4β‐OHC	/	1246	4β‐OHC was associated with lower risk of colorectal polyps	[78]
Randomised controlled trial	27‐OHC	/	1246	27‐OHC was a risk factor for colorectal adenomas	[79]
Clinical trial	/	LDLR	90	The absence of LDLR in CRC predicts a shorter survival	[80]
Randomised controlled trial	/	AMACR	725	AMACR variants were associated with advanced distal colorectal adenoma	[81]
Clinical trial	/	/	14	The cholesterol levels were rapidly decrease in CRC patients after IL‐2 treatment	[82]
Clinical trial	/	CYP7A1	355	CYP7A1 polymorphisms were associated with colorectal adenoma	[83]
A phase II study	/	/	49	The combination of simvastatin plus FOLFIRI was a feasible regimen with promising antitumour activity	[84]
A phase III study	/	/	269	The combination of simvastatin plus FOLFIRI/XELIRI did not improve PFS in CRC patients	[85]
A prospective observational study	/	/	842	The combination of simvastatin plus adjuvant chemotherapy was not associated with improved DFS, RFS, or OS in patients with stage III colon cancer	[86]

### 4.1 Cholesterol Metabolites Contribute to CRC Risk

To comprehend cholesterol metabolism's influence on CRC, it is essential to first examine how metabolic abnormalities contribute to cancer risk. A prospective study investigating the relationship between anthropometric and clinical measures linked to insulin resistance syndrome (IRS) and CRC in male smokers reported significant findings [76]. A significantly elevated risk of CRC, especially colon cancer, was observed in individuals with a BMI in the highest quintile compared to those in the lowest quintile. Furthermore, subjects presenting with a cluster of three IRS‐related conditions—hypertension, a BMI of ≥ 25 kg/m^2^ and an HDL‐C level below 40 mg/dL (1.55 mmol/L)—demonstrated a significantly higher risk of CRC compared to those with fewer such conditions [76].

In addition to these findings, cholesterol metabolites, such as oxysterols, are associated with the CRC risk. For instance, research by Passarelli et al. revealed that higher circulating levels of 7α‐hydroxycholesterol (7‐OHC) were linked to a greater risk of colorectal adenomas, whereas increased levels of 4β‐hydroxycholesterol (4β‐OHC) were associated with a reduced risk [78]. Similarly, elevated plasma concentrations of 27‐hydroxycholesterol (27‐OHC) were considerably related to an increased likelihood of developing precursor lesions for CRC [79]. These outcomes point to the crucial role of cholesterol metabolites in CRC development and hint at potential biomarkers for early intervention and detection.

### 4.2 Cholesterol Metabolism‐Associated Genes Contribute to CRC Risk

Beyond metabolic metabolites, the expression and genetic variations of cholesterol metabolism‐associated genes can also impact CRC risk. The activities of lipid transfer proteins also play a crucial role in CRC development. Specifically, the functions of lipid transfer proteins, including lecithin: LCAT and cholesteryl ester transfer protein (CETP), influence the quantity and structural integrity of HDL particles. Research has shown that CRC patients exhibit increased CETP activity alongside decreased activities of LCAT and paraoxonase‐1 [77]. Preliminary analyses suggest that CETP mass may serve as a potential indicator for CRC development, highlighting that variations in HDL‐C levels and HDL structure could be involved in the carcinogenic process [77]. Furthermore, cholesterol 7α‐hydroxylase (CYP7A1) has been proposed as a genetic modifier of CRC risk and a potential target for the treatment with bile acid, ursodeoxycholic acid (UDCA). Wertheim et al. demonstrated that CYP7A1 polymorphisms influence faecal bile acid concentrations and serve as risk factors for colorectal adenoma development, with UDCA's preventive efficacy modulated by genetic variation in CYP7A1 [83].

Genetic variations can also impact cholesterol metabolism and CRC risk. Caruso et al. explored the prognostic significance of LDLR in patients with colorectal carcinoma and found that patients lacking LDLR expression had notably shorter survival durations than those with LDLR expression [80]. Additionally, an important enzyme for cholesterol metabolite oxidation is α‐Methylacyl‐CoA racemase (AMACR), which is notably overexpressed in colorectal adenomas and cancer. A case–control study by Daugherty et al. identified specific AMACR polymorphisms associated with an elevated risk of advanced distal colorectal adenoma, underscoring the potential role of AMACR genetic variations in colorectal adenoma susceptibility [81].

### 4.3 Targeting Cholesterol Metabolism in CRC

Given the significant role of cholesterol metabolism in CRC, targeting this pathway holds promise for therapeutic interventions. In fact, the clinical significance of cholesterol metabolism in CRC extends to its therapeutic potential. For instance, IL‐2 has been shown to induce a pronounced reduction in cholesterol levels, highlighting the potential influence of cytokines on metabolic pathways beyond their immunotherapeutic functions [82].

Several clinical trials have explored the use of cholesterol‐modulating agents, such as statins, in combination therapies for CRC. Combination therapies involving statins have shown mixed results. A phase II study by Lee et al. demonstrated that combining simvastatin with conventional FOLFIRI chemotherapy in mCRC patients achieved an overall response rate (ORR) of 46.9% and a disease control rate of 83.7%, with no additional adverse effects attributed to simvastatin therapy [84]. However, a randomised phase II trial found that adding simvastatin to XELIRI/FOLFIRI chemotherapy regimens failed to improve progression‐free survival (PFS) or overall survival (OS) for mCRC patients who had undergone previous treatments [85]. Similarly, a prospective cohort study revealed that for stage III colon cancer patients, statin use during and following adjuvant chemotherapy was not linked to enhanced disease‐free survival (DFS), recurrence‐free survival (RFS), or OS [86].

In summary, cholesterol metabolism exerts a multifaceted role in CRC, influencing cancer risk, progression and therapeutic response. While genetic variations and lipid‐modulating agents offer potential therapeutic targets, clinical trials have shown mixed results regarding the efficacy of statins in CRC treatment. Further research is needed to elucidate the complex interactions between cholesterol metabolism and CRC, ultimately leading to more effective therapeutic strategies.

## 5 Conclusion and Future Perspectives

With its high incidence and mortality rates, CRC poses a significant challenge to global health [1, 2]. Recent research has stressed the critical role of cholesterol metabolism in CRC development, progression and immune interactions, offering new insights into potential therapeutic strategies [75, 87].

Cholesterol metabolism is intricately linked to CRC through multiple pathways. Elevated cholesterol levels and dysregulated lipid metabolism have been shown to promote tumorigenesis and cancer progression [88]. Specifically, cholesterol and its byproducts can alter the tumour microenvironment by affecting immune cell activity, encouraging TAM polarisation, and hindering T‐cell responses [10, 17, 89]. For example, high cholesterol levels in tumour cells can inhibit antigen presentation, enrich immunosuppressive cells, and induce CD8^+^ T cell exhaustion [40, 90]. Additionally, cholesterol metabolites such as 27‐OHC have been shown to recruit neutrophils and γδ‐T cells, thereby suppressing antitumour immunity [91].

The interaction between cholesterol metabolism and immune responses in CRC has led to the exploration of combination therapies targeting cholesterol pathways. For instance, inhibiting cholesterol biosynthesis enzymes like MVK or using statins has shown promise in enhancing antitumour immunity [88]. These strategies aim to disrupt the immunosuppressive TME and improve the efficacy of existing immunotherapies. However, clinical trials targeting cholesterol metabolism in CRC have yielded mixed results. While some studies have demonstrated improved outcomes with combination therapies, others have shown limited efficacy. This variability underscores the complexity of the TME and the need for more targeted and personalised approaches.

In the future outlook for cholesterol metabolism in CRC, several key areas should be prioritised. Elucidating molecular mechanisms is essential, as further studies are needed to fully understand how cholesterol metabolism influences CRC progression and immune evasion through specific metabolites and their interactions with immune cells. Personalised therapies should be developed to address the heterogeneity of CRC, targeting cholesterol metabolism based on individual patient characteristics such as genetic variations and tumour stage, while identifying biomarkers that predict response to these therapies. Combination strategies that integrate cholesterol metabolism inhibitors with existing treatments like chemotherapy and immunotherapy hold promise for enhancing efficacy, with preclinical and clinical studies exploring synergistic effects and optimal dosing regimens. The impact of diet and lifestyle on CRC risk and progression should also be investigated, with public health initiatives aimed at reducing high‐cholesterol diets potentially complementing medical interventions. Finally, understanding how cholesterol metabolism reshapes the TME and influences tumour‐stroma interactions could lead to novel therapeutic targets, with strategies disrupting cholesterol‐dependent TME remodelling enhancing the effectiveness of anti‐CRC therapies.

In conclusion, cholesterol metabolism is a multifaceted driver of CRC progression and immune evasion. While current research has illuminated several potential therapeutic avenues, additional research is crucial to completely utilise the clinical potential of targeting cholesterol pathways in CRC. Future efforts should focus on unravelling the complex link between cholesterol metabolism and cancer biology, ultimately facilitating more individualised and successful treatment plans.

## Author Contributions

**Chunyu Zhang:** writing – original draft (equal). **Zhiwei Miao:** writing – original draft (equal). **Yan Xu:** funding acquisition (equal), methodology (equal). **Xingchao Zhu:** supervision (equal), visualization (equal), writing – review and editing (equal). **Tongguo Shi:** supervision (equal), visualization (equal), writing – review and editing (equal).

## Ethics Statement

The authors have nothing to report.

## Consent

The authors have nothing to report.

## Conflicts of Interest

The authors declare no conflicts of interest.

## Data Availability

The data that support the findings of this study are available from the corresponding author upon reasonable request.
